# The Exposure to 2.45 GHz Electromagnetic Radiation Induced Different Cell Responses in Neuron-like Cells and Peripheral Blood Mononuclear Cells

**DOI:** 10.3390/biomedicines11123129

**Published:** 2023-11-24

**Authors:** Maria Paola Bertuccio, Giuseppe Acri, Riccardo Ientile, Daniela Caccamo, Monica Currò

**Affiliations:** Department of Biomedical and Dental Sciences and Morpho-Functional Imaging, University of Messina, 98125 Messina, Italy; giuseppe.acri@unime.it (G.A.); ientile@unime.it (R.I.); daniela.caccamo@unime.it (D.C.); monica.curro@unime.it (M.C.)

**Keywords:** electromagnetic radiation, oxidative stress, mitochondrial activity, neuronal-like cells, peripheral blood mononuclear cells

## Abstract

Electromagnetic radiation emitted by commonly used devices became an issue for public health because of their harmful effects. Notably, 2.45 GHz electromagnetic radiation exposure has been associated with DNA damage and alterations in the central nervous system. We here investigated the effects of 2.45 GHz electromagnetic radiation on cell redox status by using human SH-SY5Y neuroblastoma cells, which were differentiated to neuronal-like cells, and peripheral blood mononuclear cells (PBMCs), which were exposed to an antenna emitting 2.45 GHz electromagnetic radiation for 2, 24, and 48 h. We evaluated cell viability and mitochondrial activity alterations by measuring reactive oxygen species (ROS), mitochondrial transmembrane potential (ΔΨm), NAD+/NADH ratio, mitochondrial transcription factor A (*mtTFA*), and superoxide dismutase 1 (*SOD1*) gene transcript levels. We also investigated apoptosis and autophagy, evaluating B-cell lymphoma 2 (*BCL2*), BCL2-associated X protein (*BAX*), and microtubule-associated protein 1A/1B-light chain 3 (*LC3*) gene transcript levels. Cell viability was significantly reduced after 24–48 h of exposure to radiation. ROS levels significantly increased in radiation-exposed cells, compared with controls at all exposure times. ΔΨm values decreased after 2 and 24 h in exposed SH-SY5Y cells, while in PBMCs, values decreased soon after 2 h of exposure. Alterations were also found in the NAD+/NADH ratio, *mtTFA*, *SOD1*, *LC3* gene expression, and *BAX/BCL2* ratio. Our results showed that neuron-like cells are more prone to developing oxidative stress than PBMCs after 2.45 GHz electromagnetic radiation exposure, activating an early antioxidant defense response.

## 1. Introduction

In the last thirty years, there has been an ever-increasing use of electronic devices, such as cell phones, communication base stations, high-voltage lines, electronic instruments, and microwave ovens. This has led to the onset of the so-called electromagnetic smog, due to the electromagnetic radiation (EMR) emitted by these devices. Radio communications between mobile phones and transmitter–receptor antenna were performed using radiofrequency electromagnetic fields (RF-EMF) in the frequency ranging from 900 to 1800 MHz [1].

The fourth-generation (4G) long-term evolution (LTE) is designed to work across a number of frequency bands currently ranging from 450 MHz up to 3.8 GHz. In our research project, we chose to expose the cells to a selected frequency of 2.45 GHz, as it falls within the range of the frequency band assigned for industrial, scientific, medical, and domestic (ISMD) applications and for many wireless communication standards [2].

Several studies evaluated the impact of EMR on human health, and correlations have been found between exposure to radiation and the onset of various diseases, such as brain tumors, childhood leukemia, male infertility, and neurodegenerative disease [3,4,5,6]. EMR can pass through human body tissues, influencing cellular biochemical processes and altering the cell membrane potential and the distribution of ions and dipoles [7,8]. Several factors influence molecular and cellular alterations induced by radiation, such as the duration of radiation exposure, heat generated, and degree of tissue permeability [1,9].

One of the effects of EMR on biological systems can be the increase in free radicals, with consequent lipid peroxidation and alteration of antioxidant activities, given that oxidative stress can be considered a key factor in the onset of harmful effects linked to radiation exposure [3,8]. Nevertheless, the involvement of oxidative stress in EMR-related pathologies has generated conflicting results. A possible cause of this may be the different redox responses of the cell lines used for these researches [10].

Several studies have found no effects of RF exposure on ROS or genotoxic effects in human lymphocytes using electromagnetic radiation lower than 2.0 GHz [4,11]. On the contrary, other in vivo and in vitro studies found that the electromagnetic field emitting from mobile phones might induce ROS formation in different organs and tissues. In fact, exposure to EMR can cause detrimental effects on the central nervous system, the cardiovascular system, and the hematopoietic and reproductive systems [8,9,12,13,14].

Mobile phone use and 2.45 GHZ exposure have been associated with DNA damage and the related alterations in the central nervous system, including increased permeability of the blood–brain barrier (BBB), increase in calcium ion efflux, depressed activities of different types of neurotransmitter systems, cognitive disfunction, and sleep disorders [15,16].

Given the correlations found between brain tumors, alterations in cognitive and physiological functions, and RF radiation [17,18,19,20,21,22], we aimed to investigate the onset of oxidative stress and the alteration of mitochondrial permeability in SH-SY5Y neuroblastoma cells differentiated to dopaminergic neuron-like cells after brief and extended exposures to RF-EMF in 2.45 GHz frequencies. The SH-SY5Y neuroblastoma cells differentiated into neuronal-like cells with retinoic acid represent a widely employed model used in neurobiology studies. Differentiated cells present long, branched processes and a decreased proliferation rate, and they express different markers of mature neurons, as reported by Shipley et al., 2016 [23].

It has been reported that exposure to RF-EMF could cause genetic toxic effects in blood lymphocytes [24]. Radiation emitted from mobile base stations have been linked to an increase in DNA strand breaks in peripheral blood leukocytes of mobile phone users and individuals residing in proximity of a mobile base [25].

Furthermore, considering that blood is exposed more than other tissues to environmental electromagnetic fields, as it is spread in every part of the body under the skin, we analyzed the alterations induced in peripheral blood mononuclear cells (PBMCs) in terms of oxidative stress and mitochondrial functionality after brief and extended exposures to 2.45 GHz radiation.

To our knowledge, there are no previous studies about the effects of the exposition of dopaminergic neuron-like cells and PBMCs to 2.45 GHz frequency radiation on mitochondrial functionality.

## 2. Materials and Methods

The SH-SY5Y cell line (CRL-2266) was obtained from the American Type Culture Collections (ATCC) (Rockville, MD, USA). The following reagents were purchased from SIGMA Aldrich (Milan, Italy): fetal bovine serum (FBS), antibiotics, minimum essential medium (MEM) eagle (M5650), Nutrient Mixture F-12 Ham (M4888), RPMI-1640 medium, all-trans retinoic acid (RA), sodium pyruvate, and phosphate-buffered saline (PBS) solution.

### 2.1. Cell Culture and Treatment

SH-SY5Y cells were cultured in 6-well plates at a density of 5⋅10^5^ cell/mL using a medium containing an equal quantity of MEM and Ham’s F-12 (1:1), in which 10% (*v*/*v*) heat-inactivated FBS, L-glutamine (2 mM), and sodium pyruvate (1 mM) were added. The plates were placed inside an incubator with 5% CO_2_ and 95% air humidity at a temperature of 37 °C.

When sub-confluent cells were washed two times with PBS, they were subsequently incubated in MEM/Ham’s F-12 medium containing 1% FBS, L-glutamine (2 mM), and sodium pyruvate (1 mM) for 24 h. Suddenly, the medium was replaced with a fresh one containing the addition of 10 μM RA (10 mM in dimethyl sulphoxide (DMSO) stock solution). The medium was renewed every two days. After 5 days of 10 μM RA exposure, differentiated SH-SY5Y cells were exposed to the electromagnetic field [26].

### 2.2. Blood Sample Collection and PBMC Culture

The blood samples were collected by venipuncture from volunteers and placed in individual heparinized tubes. PBMC separations were performed using Histopaque^®^-1077 (Sigma, Milan, Italy) following the manufacturer’s instructions. PBMC suspensions were washed twice with PBS, collected by centrifugation, and then suspended at a density of 4 × 10^6^ cells/well in 6-well plates containing RPMI-1640 medium supplemented with 10% fetal calf serum and 1% antibiotics (100 IU/mL penicillin and 100 mg/mL streptomycin). The cultures were incubated at 37 °C in a humidified 5% CO_2_ atmosphere for 24 h and then exposed to the electromagnetic field.

### 2.3. Experimental Design

The experimental set-up used to generate electromagnetic waves consisted only of a signal generator Hewlett—Packard 8648C (Agilent Technologies, Santa Clara, CA, USA) working in the frequency range of 100–3200 MHz, with an output RF level ranging from –136 dBmW to + 20 dBmW, and a biconical transmitting antenna (RKB) working in the frequency range of 1.7–2.5 GHz and having an isotropic gain of about 0 dBmW in the same frequency range, all connected by a cable.

We set an output power of 0 dB (mW) corresponding to 1 mW and a frequency of 2.45 GHz.

**Figure 1:**
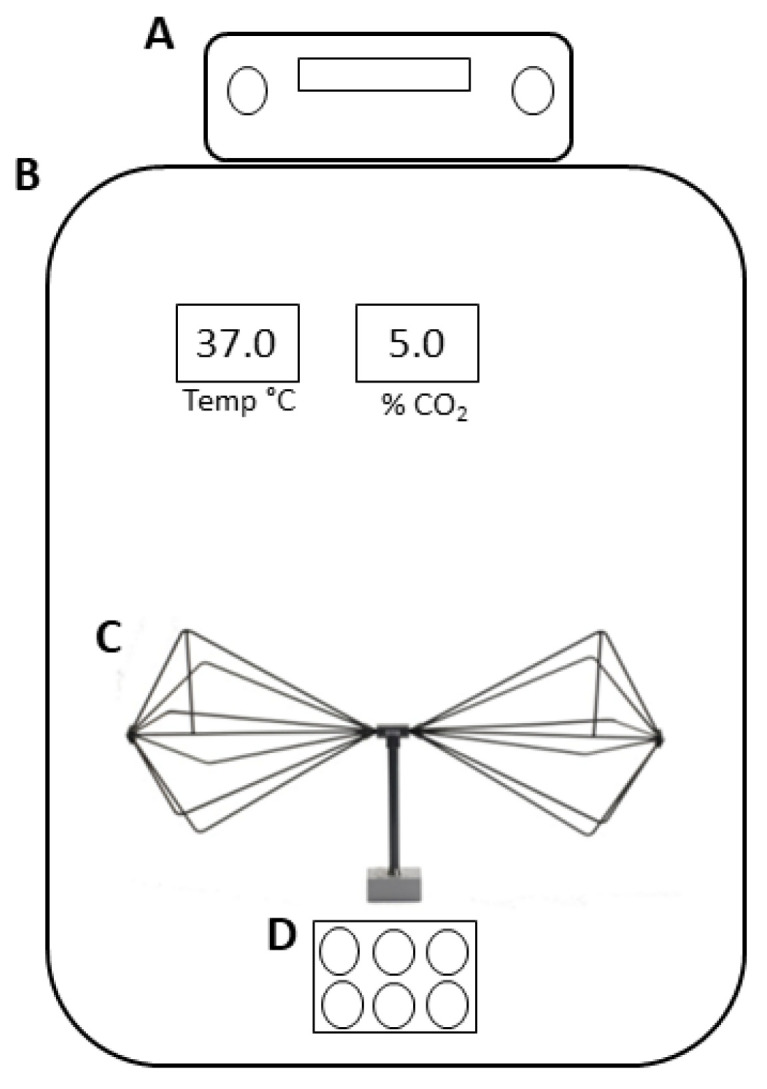
Schematic representation of the EMF exposure system. (**A**) Signal generator, (**B**) incubator, (**C**) biconical transmitting antenna, and (**D**) 6-well plate.

As shown in Figure 1, the antenna was placed inside an incubator, which was maintained at a temperature of 37 °C, 5% CO_2_, and 95% air humidity (incubator series 5400-115V models, Thermo Electron Corporation, Winchester, VA, USA).

Before starting the study, we conducted measurements to test the exposure system. The electromagnetic field (EMF) exposures were evaluated by using the PMM 5083-b (Narda Safety Test Solutions, Cisano sul Neva (Savona)—Italy) portable field meter, coupled with a triaxial electric field probe EP745 (frequency range 100–7 GHz and dynamic range 0.35–450 V/m). These measurements were divided into different steps, consisting of:

- The measurement of EMF out of the incubator with the signal generator switched off;
- The measurement of EMF out of the incubator with the signal generator switched on;
- The measurement of EMF inside the incubator with the signal generator turned off and the electric field probe positioned at 20 cm from the source element antenna;
- The measurement of EMF inside the incubator with the antenna turned on and the electric field probe positioned at 20 cm from the source element antenna.

RA-differentiated SH-SY5Y cells and PBMCs grown in 6-well plates were exposed to the electromagnetic field for 2, 24, and 48 h. The plates were placed at the center of a uniform field area under the antenna and at a distance of approximately 10 cm from the antenna element, the source of the electromagnetic field. This distance was necessary to avoid the heat produced by the device.

Cells not exposed to radiation were placed into a second incubator of the same model of the one cited above, maintaining the same physical conditions previously reported.

To verify whether the electromagnetic field heated the sample, we carried out temperature measurements by using a digital immersion thermometer (Testo 735, Alton Hampshire—UK), measuring the range from −80 °C to 300 °C. The measurements were conducted inside the incubator,

(a) with the signal generator antenna turned off and (b) with the signal generator turned on.

The temperature probe was positioned inside the sample under the antenna. The duration of measurements was 48 h. In both cases, there was no increase in sample temperature.

### 2.4. Cell Viability Assay

Cell viability was measured by a quantitative colorimetric assay with 3-[4,5-dimethylthiazol-2-yl]-2,5-diphenyltetrazolium bromide (MTT). SH-SY5Ycells were cultured in 96-well culture plates at a density of 5 × 10^4^ cell/well, differentiated with RA, and exposed to the electromagnetic field. PBMCs were instead incubated in 96-well culture plates at a density of 1 × 10^4^ cells per well.

After treatment, the cells were washed and incubated with fresh red phenol-free medium containing MTT (0.5 mg/mL) at 37 °C for 4 h. Then, insoluble formazan crystals were dissolved in 100 µL of a 0.04 N HCl/isopropanol solution at 37 °C for 1 h. The optical density in each well was evaluated by a spectrophotometrical measurement of absorbance at 570 nm using a microplate reader (Tecan Italia, Cologno Monzese, Italy).

All experiments were performed in quintuplicate. The results were showed as the percent of cell viability of the exposed groups to the respective untreated samples.

### 2.5. Measurement of Intracellular Reactive Oxygen Species

Intracellular reactive oxygen species (ROS) production was quantified by fluorescence with 2′,7′-dichlorofluorescein diacetate (H2DCF-DA), which reacts with ROS to generate the fluorescent product 20-70-dichlorofluorescein (DCF). At the end of each treatment, RA-differentiated SH-SY5Y cells were incubated with 5 μM H2DCF-DA and dissolved in dimethylsulfoxide (DMSO) for 30 min at 37 °C in the dark. Then, SH-SY5Y cells were washed twice with PBS (pH 7.4), harvested with non-enzymatic cell dissociation solution, and resuspended in 500 mL of PBS supplemented with 0.1 M KH_2_PO_4_ and 0.5% Triton X-100. After centrifugation at 13,000 rpm for 10 min, supernatants were collected, and the fluorescence intensity was analyzed at an excitation wavelength of 480 nm and an emission wavelength of 540 nm by a microplate reader (Tecan Italia, Cologno Monzese, Italy).

PBMCs were incubated with 5 μM H2DCF-DA, dissolved in dimethylsulfoxide (DMSO) for 30 min at 37 °C, washed twice with PBS (pH 7.4), and resuspended in 500 µL of PBS supplemented with 0.1 M KH_2_PO_4_ and 0.5% Triton X-100. After centrifugation at 13,000 rpm for 10 min, supernatants were collected, and the fluorescence intensity was analyzed as described above.

Cell lysates were analyzed for protein content using the Lowry method, and DCF fluorescence was normalized for total protein content. All experiments were performed in triplicate.

### 2.6. Measurement of Mitochondrial Transmembrane Potential (ΔΨm)

Alterations in mitochondrial transmembrane potential (ΔΨm) were assayed by the incorporation of a cationic fluorescent dye rhodamine 123 (Rh-123). After the treatments previously described, 10 μM Rh-123, dissolved in DMSO, was added to the fresh mediums of stimulated and unstimulated cells (SH-SY5Y and PBMC) and incubated for 30 min at 37 °C in the dark. The cells were then collected and washed twice with PBS (pH 7.4), and the fluorescence intensity was analyzed at wavelengths of 488 nm for excitation and 525 nm for emission by a microplate reader (Tecan Italia, Cologno Monzese, Italy). Cell lysates were analyzed for protein content using the Lowry method, and rhodamine 123 fluorescence was normalized for total protein content. All experiments were performed in triplicate.

### 2.7. Measurement of NAD+/NADH Ratio

The NAD+/NADH ratio amount in differentiated SH-SY5Y cells and PBMCs was determined using the AmpliteTM Colorimetric NAD/NADH Assay Kit (AAT BioQuest, Sunnyvale, CA, USA) according to the manufacturer’s instructions.

The color reaction of the prepared samples was read at 450 nm using a microplate reader (Tecan Italia, Cologno Monzese, Italy). All experiments were performed in triplicate.

### 2.8. Real-Time PCR

To isolate total RNA from cells under study, the Trifast reagent was used. The reverse transcription of two micrograms of RNA was carried out with the High-Capacity cDNA Archive kit according to the manufacturer’s instructions. Afterwards, mRNA levels of *mtTFA, SOD1, BAX, BCL2*, and *LC3* were analyzed by real-time PCR using SYBR green-based gene expression analysis. We chose to use β-actin as the endogenous control. Primer sequences used and the lengths of expected products are listed in Table 1.

Quantitative PCR reactions were executed in a solution of 1 X SYBR green PCR Master mix, 0.1 μM specific primers, and 25 ng cDNA in a final volume of 10 μL. A 96-well plate was used to perform the experiments in triplicate in a 7900HT Fast Real-Time PCR System (Applied Biosystems, Foster City, CA, USA) with the following set up: 1 cycle at 95 °C for 10 min, followed by 45 cycles at 95 °C for 15 s, and then 60 °C for 1 min. A standard dissociation stage was added to assess the primer annealing specificity. To determine the relative expression of the genes under study, we analyzed the obtained data using SDS 2.3 and RQ manager 1.2 software (Applied Biosystems, Foster City, CA, USA) based on the 2^−ΔΔCT^ relative quantification method.

### 2.9. Statistical Analysis

All values are expressed as mean ± standard error of the mean (SEM). Statistical analysis was carried out using Student’s *t*-test for comparisons between two groups, with *p*-values less than 0.05 considered significant.

## 3. Results

The results obtained from electromagnetic background measurements (steps a and c indicated in Section 2.3) showed values < 0.35 V/m in both cases. The measurements referred to in steps b and d showed an electric field value of 1.8 ± 0.05 V/m in both cases, excluding any interference caused by metal inside the incubator.

**Figure 2:**
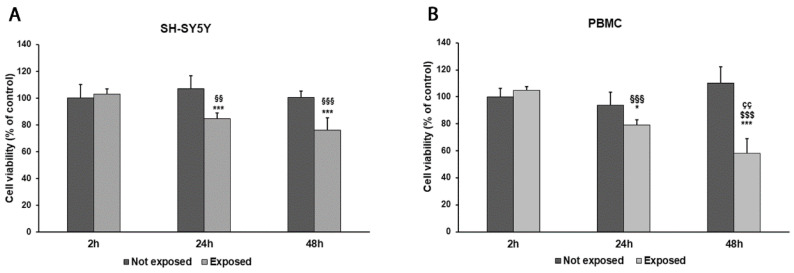
Cell viability was assessed by MTT assay in differentiated SH-SY5Y cells (**A**) and in PBMCs (**B**) exposed and not exposed to 2.45 GHz radiation. * *p* < 0.05 and *** *p* < 0.001: significant differences between exposed vs. not exposed; §§ *p* < 0.01 and §§§ *p* < 0.001: significant differences between exposed for 24 and 48 h vs. exposed for 2 h; $$$ *p* < 0.001: significant difference between exposed for 48 vs. exposed for 2 h; çç *p* < 0.01: significant difference between exposed for 48 h vs. exposed for 24 h.

While comparing early vs. late exposure times, we noticed a progressive reduction in cell viability in both SH-SY5Y cells and PBMCs. Indeed, the exposure of differentiated SH-SY5Y cells and PBMCs for 2 h to 2.45 GHz did not significantly affect cell viability in comparison to non-irradiated control cells. Instead, SH-SY5Y cell viability was reduced by 20% after 24 h (*p* = 0.0018) and by 24% after 48 h (*p* < 0.0001). Similarly, cell viability in PBMCs was reduced by 16% after 24 h of exposure (*p* = 0.0120) and by 47% after 48 h (*p* = 0.0001), compared with control cells (Figure 2A,B). However, no changes in cell morphology were observed after exposure.

**Figure 3:**
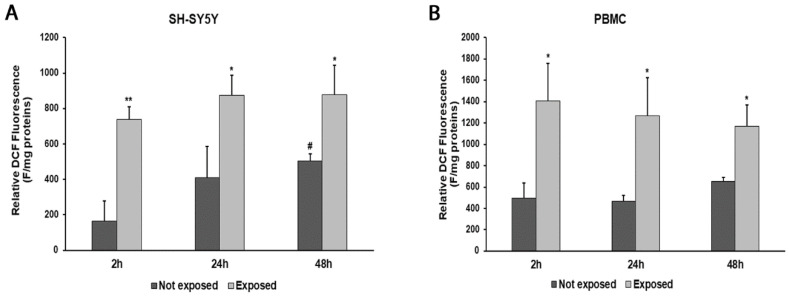
ROS production in differentiated SH-SY5Y cells (**A**) and in PBMCs (**B**) exposed and not exposed to 2.45 GHz radiation. * *p* < 0.05 and ** *p* < 0.01: significant differences between exposed vs. not exposed; # *p* < 0.05: significant differences between not exposed for 48 h vs. not exposed for 2 h.

An increase in ROS production was observed at all times of exposure to 2.45 GHz radiation in both SH-SY5Y cells and PBMCs, compared with control cells (Figure 3A,B). In particular, ROS levels increased by 346% (*p* = 0.0017) after 2 h, 112% (*p* = 0.0190) after 24 h, and 75% (*p* = 0.0193) after 48 h in SH-SY5Y cells exposed to radiation, compared with their unexposed counterpart. Instead, ROS production increased by 183% (*p* = 0.0143) after 2 h, 170% (*p* = 0.0194) after 24 h, and 78% (*p* = 0.0116) after 48 h in PBMCs exposed to radiation, compared with those not exposed.

**Figure 4:**
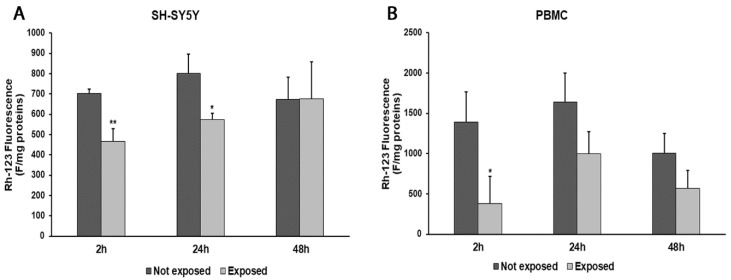
Changes in ΔΨm in differentiated SH-SY5Y cells (**A**) and in PBMCs (**B**) exposed and not exposed to 2.45 GHz radiation. * *p* < 0.05 and ** *p* < 0.01: significant differences between exposed vs. not exposed.

In parallel, consistent with ROS changes, we observed a decrease in ΔΨm values after 2 and 24 h in radiation-exposed SH-SY5Y cells, compared to unexposed cells (33%, *p* = 0.0036, and 28%, *p* = 0.0182, respectively). Instead, after a longer exposure time, no difference in the ΔΨm values was found (Figure 4A). In PBMCs exposed to radiation, we obtained ΔΨm value reductions of 73% (*p* = 0.0247) after 2 h of exposure, 39% after 24 h, and 43% after 48 h in comparison with unexposed cells (Figure 4B).

**Figure 5:**
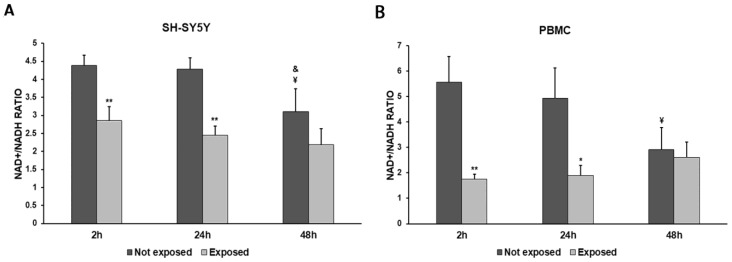
NAD+/NADH ratio in differentiated SH-SY5Y cells (**A**) and in PBMCs (**B**) exposed and not exposed to 2.45 GHz radiation. * *p* < 0.05 and ** *p* < 0.01: significant differences between exposed vs. not exposed; ¥ *p* < 0.05: significant difference between not exposed for 48 h vs. not exposed for 2 h; & *p* < 0.05: significant difference between not exposed for 48 h vs. not exposed for 24 h.

**Figure 6:**
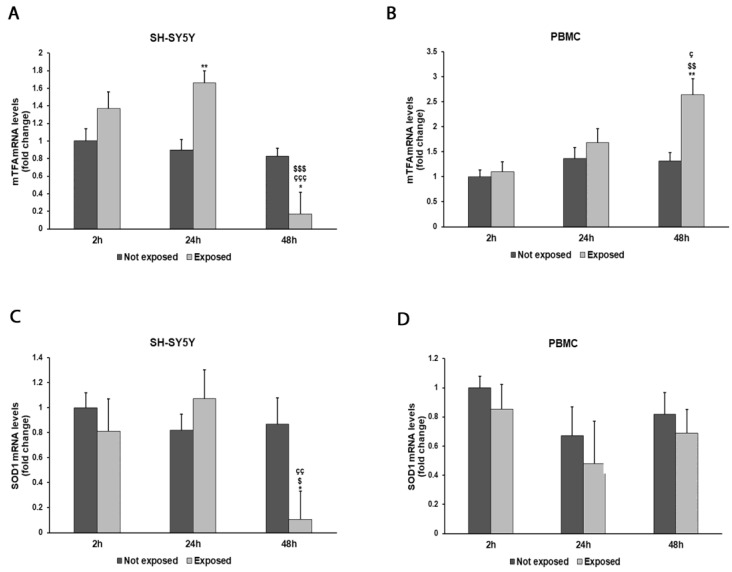
Effect of 2.45 GHz radiation on the *mtTFA* gene in differentiated SH-SY5Y cells (**A**) and in PBMCs (**B**) and on the *SOD1* gene in differentiated SH-SY5Y cells (**C**) and in PBMCs (**D**). The mRNA transcript levels were analyzed by real-time-PCR. * *p* < 0.05, ** *p* < 0.01: significant differences between exposed vs. not exposed; $ *p* < 0.05, $$ *p* < 0.01, and $$$ *p* < 0.001: significant differences between exposed for 48 h and exposed for 2 h; ç *p* < 0.05, çç *p* < 0.01, and ççç *p* < 0.001: significant differences between exposed for 48 h and exposed for 24 h.

In order to more deeply investigate the effects of the exposure to 2.45 GHz radiation on mitochondrial dynamics in SH-SY5Y cells and PBMCs, we evaluated the NAD+/NADH ratio (Figure 5A,B) and the expressions of *mtTFA* and *SOD1* genes, coding for proteins involved in the redox status of cultured cells (Figure 6A–D).

NADH consumption was observed in both cell types. The exposure to 2.5 GHz radiation produced a decrease in the NAD+/NADH ratio in exposed cells, compared to not-exposed cells, especially after 2 h and 24 h, while this difference became smaller after 48 h. The NAD+/NADH ratio was decreased by 35% after 2 h (*p* = 0.0055), by 43% after 24 h (*p* = 0.0015), and by 29% after 48 h (*p* = 0.1078) in SH-SY5Y cells exposed to radiation, compared with unexposed cells. Greater differences in the NAD+/NADH ratio between exposed and unexposed cells were observed in PBMCs. In fact, we found a 69% decrease after a 2 h exposure (*p* = 0.0030), a 62% decrease after a 24 h exposure (*p* = 0.0140), and an 11% decrease after a 48 h exposure (*p* = 0.5872).

The expressions of *mtTFA* and *SOD1* genes showed variations associated with cellular exposure to electromagnetic radiation (Figure 6A–D). In particular, *mtTFA* expression increased by 84% in SH-SY5Y cells after a 24 h exposure to radiation (*p* = 0.0020) and by 100% in PBMCs after a 48 h exposure (*p* = 0.0032) in comparison with unexposed cells. Instead, an 88% decrease in *SOD1* expression was observed after a prolonged exposure in SH-SY5Y cells (*p* = 0.0131), while no significant variations were observed in PBMCs.

**Figure 7:**
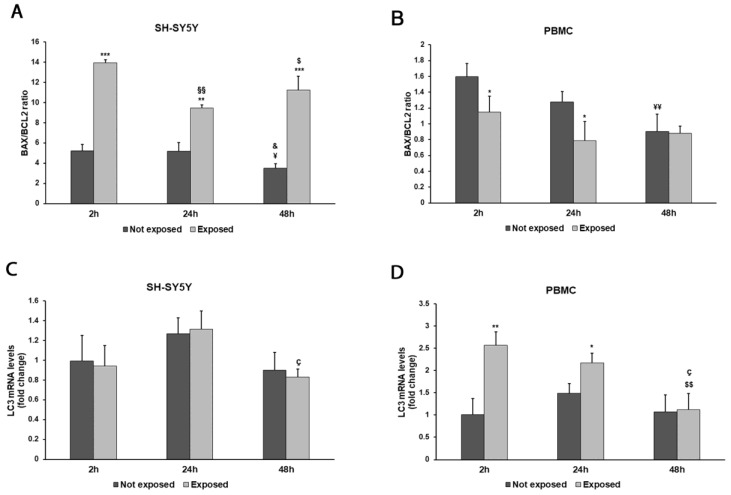
Effect of 2.45 GHz radiation on the *BAX/BCL2* ratio in differentiated SH-SY5Y cells (**A**) and in PBMCs (**B**) and on the *LC3* gene in differentiated SH-SY5Y cells (**C**) and PBMCs (**D**). The mRNA transcript levels were analyzed by real time-PCR. * *p* < 0.05 ** *p* < 0.01, and *** *p* < 0.001: significant differences between exposed vs. not exposed; §§ *p* < 0.01: significant difference between exposed for 24 and 48 h vs exposed for 2 h; $ *p* < 0.05 and $$ *p* < 0.01: significant differences between exposed for 48 h and exposed for 2 h; ç *p* < 0.05: significant difference between exposed for 48 h and exposed for 24 h; ¥ *p* < 0.05 and ¥¥ *p* < 0.01: significant differences between not exposed for 48 h vs. not exposed for 2 h; & *p* < 0.05: significant difference between not exposed for 48 h vs. not exposed for 24 h.

To investigate the impact of radiation on cellular apoptosis and autophagy, we evaluated gene transcript levels of the pro- and anti-apoptotic markers BAX and BCL-2 and of the autophagy marker light chain 3 (*LC3*) (Figure 7A–D).

The exposure to 2.45 GHz radiation enhanced apoptosis, even after a brief exposition in differentiated SH-SY5Y cells, to a great extent after 48 h (*p* = 0.0008), as shown by the ratio of BAX/BCL-2 mRNA levels (Figure 7A). On the contrary, in PBMCs, we did not find pro-apoptotic behaviour (Figure 7B), while these cells were more prone to autophagy after exposition for 2 and 24 h (*p* = 0.0044 and *p* = 0.0193, respectively) (Figure 7D).

## 4. Discussion

The increased use of electronic devices in daily life, with a parallel increased number of adverse effects caused by EMR, gives rise to many concerns about their effects on human health. It is known that EMR in the range of 2.45 GHz generates significant DNA damage in mice models [27]. A study conducted by Ömer Çelik et al. reported that EMR exposure may lead to excessive ROS production and reduced antioxidant defense systems, resulting in brain and liver damage and the degradation of membranes during the pregnancy and development of rat newborns [28]. According to Vijayalaxmi’s cytogenetic study on human blood lymphocytes exposed to 2.45 GHz of RF radiation, a 2 h exposure of the cells to this radiation does not cause genotoxic effects; however, the International Agency for Research on Cancer (IARC) considers EMF and extremely-low-frequency (ELF) MF as possibly carcinogenic to humans (Group 2B) [29,30]. As reported in Schuermann and Mevissen’s review, several studies conducted on animals and cells showed increased oxidative stress caused by RF-EMF and ELF-MF [31]. Altered ROS levels can affect biochemical and signaling processes, resulting in nucleic acid and protein oxidative damage and fatty acid peroxidation, leading to vital cellular process and function alterations, such as cell proliferation, differentiation, and inflammation [32,33].

Because of EMF controversy, the World Health Organization (WHO) is conducting systematic reviews about this topic that hopefully will improve the validity of future research on the exposure effects of non-ionizing energy [34].

Our results showed a time-dependent decrease in cell viability in comparison to unexposed cells; in particular, we observed pro-apoptotic events in neuronal-like cells, while the activation of the autophagic pathway was found in PBMCs. A previous study conducted by Ozgur et al. investigating the alteration of proliferation in hepatocarcinoma cells induced by mobile phone radiation reported that the frequency of the applied field and the exposure duration modify cell viability [35]. Previous results of a study by Esmekaya et al. showed an inhibitory effect of RF radiation on lymphocyte viability that was marked during longer exposure periods [36]. Research by Gulati et al. confirmed the presence of a decrease in lymphocyte viability when exposed to UMTS, but in this case, it was caused by endogenous apoptosis and not by the exposure [4]. In a review on the effects of RF-EMF exposure in cultured mammalian, the authors Manna and Gosh (2016) evaluated apoptosis and several other biological outcomes; they concluded that RF-EMF exposure might affect the apoptotic process in vitro, and the results depend on the cell model used and on the experimental design [37].

To investigate possible causes of cell alterations after EMF exposure, we analyzed ROS production and mitochondrial functionality, with mitochondria being a major source of ROS and also the main target of their detrimental effects. Several studies, have shown the induction of oxidative stress of RFR exposure under different conditions, as indicated by the increase in ROS, peroxidized lipids, oxidized proteins, and fragmented DNA [38,39,40]. We found an increase in ROS production, especially after the first few hours of radiation exposure, compared to unexposed cells; the exposure might induce ROS production temporarily due to subsequent stimulation of the antioxidant defense mechanism [41,42]. Indeed, the amount of the ROS elevation after exposure to EMF could be associated with the decrease in mitochondrial potential, as previously suggested by Santini et al., 2018. Although other studies have obtained conflicting results regarding the effect of this exposure on mitochondrial potential, this could depend on the use of different experimental models and the intensity of the field used [43]. We obtained similar results in the two different kinds of cells evaluated after expositions of 2 and 24 h, while after 48 h of treatment, mitochondrial potential continued to decrease in PBMCs but not in SH-SY5Y cells, in which we noticed a restoration to their original mitochondrial functionality. The variability in cell behavior after different time exposures may be due to the activation of adaptative mechanisms that occur after earlier cellular events.

Mitochondria play a critical role for NAD+NADH metabolism, and the involvement of NAD+ in the tricarboxylic acid cycle and NADH in the electron transport chain underscore the importance of maintaining an optimal NAD+/NADH ratio to mitochondrial function [44].

Assuming the induction of a slowdown at the level of the electron transport chain, we evaluated the NAD+/NADH ratio and found a decrement with respect to unexposed cells, especially after an early exposition. Moreover, we also noticed a gradual decrease in the NAD+/NADH ratio over time in unexposed cells, probably due to the fact that, as reported by Yang and Sauve, under normal cellular conditions, the synthesis of NAD+ is affected by the availability of possible precursors, so availabilities of nicotinic acid, nicotinamide riboside, nicotinamide, and tryptophan can alter NAD+ synthetic rates and the NAD+/NADH ratio as a consequence [45]. Previous observations are useful to explain that the values of NAD+/NADH reported here reflect changes in oxidative metabolism.

The antioxidant enzyme alteration is linked to the decline in mitochondrial potential. It is not new that the exposure to electromagnetic fields influences the transcriptional and protein levels of antioxidant genes, such as *SOD1, SOD2, CAT*, and *GPx1* in several cell lines, leading to their downregulation [46,47,48,49].

Parallel to the decrease in mitochondrial potential, EMR exposure was also related to an increase in damages to the mitochondrial DNA, along with a reduction in its copy number and mitochondrial RNA transcription level [50,51,52,53]. For this reason, we chose to evaluate the expressions of *SOD1* and *mtTFA*, the last ones required for the replication of mtDNA, regulating the number of mtDNA copies. The increase in *mtTFA* expression was time dependent in PBMCs, while it showed a sudden decline in the transition from 24 to 48 h of exposure in SH-SY5Y cells; this may be related to the different resilience of the two cell lines as also shown by the mitochondrial membrane potential results discussed above. Significant reductions in the levels of the antioxidant gene *SOD1* became evident after a prolonged exposure in SH-SY5Y cells, with these results being correlated with exposure duration, as also reported in a review by Schuermann and Mevissen, 2021 [31].

In PBMCs, no significant reduction in *SOD1* expression was detected, in accordance with the study of Asl et al., 2020, which analyzed the RF-EMF exposure of male Wistar rats for 1 h/day for 1 month, resulting in increased oxidative stress, NO formation, and reduced antioxidant markers [54]. The reason for the weak effect of radiation on *SOD1* expression in PBMCs in comparison to SH-SY5Y cells can be explained by the fact that stress responses might be different depending on cell type and experimental conditions, and based on our results, it seems that neuronal cells might react in a stronger way with respect to peripheral cells activating early antioxidant enzymatic activity.

## 5. Conclusions

Taken together, the current research results showed that 2.45 GHz EMR emitted from commonly used devices is capable of influencing both ROS production and mitochondrial imbalance, but it must be considered that these effects may vary based on the experimental parameters. In fact, they may depend on the time of exposure to electromagnetic fields and on the kind of cells used. Therefore, to protect individuals from the adverse effects of EMR exposure on the antioxidant potential of our cells, the use of such devices should be limited. Further investigations are necessary to better understand and confirm these observations, taking into account the complexity of the immune network and increasing the radiation exposure time.

## Figures and Tables

**Table 1:** Primers used for real-time PCR analysis of gene expression and lengths of expected products.

Target	Primer Sequence 5′ > 3′		Length of Expected Product (bp)
	Forward	Reverse	
*β-actin*	TTGTTACAGGAAGTCCCTTGCC	ATGCTATCACCTCCCCTGTGTG	100
*mtTFA*	TCATCTGTCTTGGCAAGTTGTC	AGTCCGCCCTATAAGCATCT	192
*SOD1*	GGTGTGGCCGATGTGTCTATT	CTGCTTTTTCATGGACCACCA	73
*LC3*	CGGTGATAATAGAACGATACAAG	CTGAGATTGGTGTGGAGAC	186
*BAX*	GGACGAACTGGACAGTAACATGG	GCAAAGTAGAAAAGGGCGACAAC	127
*BCL2*	ATCGCCCTGTGGATGACTGAG	CAGCCAGGAGAAATCAAACAGAGG	105

## Data Availability

The raw data supporting the conclusions of this article will be made available by the authors upon reasonable request.
